# Development and validation of a survival model for thyroid carcinoma based on autophagy-associated genes

**DOI:** 10.18632/aging.103715

**Published:** 2020-10-14

**Authors:** Baoai Han, Xiuping Yang, Davood K. Hosseini, Pan Luo, Mengzhi Liu, Xiaoxiang Xu, Ya Zhang, Hongguo Su, Tao Zhou, Haiying Sun, Xiong Chen

**Affiliations:** 1 Public Laboratory, Key Laboratory of Breast Cancer Prevention and Therapy, Ministry of Education, Tianjin Medical University Cancer Institute and Hospital, National Clinical Research Center for Cancer, Tianjin Medical University, Tianjin 30000, China; 2 Department of Otorhinolaryngology, Head and Neck Surgery, Zhongnan Hospital of Wuhan University, Wuhan 430071, China; 3 Department of Otolaryngology-Head and Neck Surgery, Stanford University School of Medicine, Stanford, CA 94305, USA; 4 Department of Internal Medicine, Stanford University School of Medicine, Stanford, USA 94305, USA; 5 Department of Otorhinolaryngology, Head and Neck surgery, Wuhan Central Hospital, Wuhan 430014, China; 6 Department of Otorhinolaryngology, Union Hospital, Tongji Medical College, Huazhong University of Science and Technology, Wuhan 430022, China

**Keywords:** thyroid carcinoma, autophagy-related genes, TCGA database, prognosis

## Abstract

Abnormalities in autophagy-related genes (ARGs) are closely related to the occurrence and development of thyroid carcinoma (THCA). However, the effect of ARGs on the prognosis of THCA remains unclear. Here, by analyzing data from TCGA, 26 differentially expressed ARGs were screened. Cox regression and Lasso regression were utilized to analyze the prognosis of the training group, and a risk model was constructed. Our results show that low-risk patients had better overall survival (OS) than high-risk patients, and the area under the ROC curve in the training and testing groups was significant (3-year AUC, 0.735 vs 0.796; 5-year AUC, 0.821 vs 0.804). In addition, a comprehensive analysis of the 5 identified ARGs demonstrated that most of them were related to OS in THCA patients, and two of them (CX3CL1 and CDKN2A) were differentially expressed in THCA and normal thyroid tissues at the protein level. GSEA suggested that the inactivation of the cell defense system and the activation of some classical tumor signaling pathways are important driving forces for the progression of THCA. This study demonstrated that the 5 ARGs in the survival model are promising multidimensional biomarkers for the diagnosis, prognosis, and treatment of THCA.

## INTRODUCTION

Thyroid carcinoma is the most common malignant tumor of the endocrine system, accounting for 4% of all new tumors and ranking fifth among female patients, with a high incidence [1, 2]. Since the mid-1990s, its incidence has increased year by year. Thyroid carcinoma seriously affecting the physical and mental health of patients [3, 4]. According to histopathology and clinical characteristics, thyroid carcinoma can be divided into 4 types: papillary, follicular, medullary, and undifferentiated thyroid carcinoma [5]. At present, surgery combined with radioiodine therapy, radiation therapy or chemotherapy is mostly used in the treatment of thyroid cancer. However, for radiation-resistant papillary thyroid cancer, undifferentiated thyroid cancer, and medullary thyroid cancer, these treatment regimens cannot provide complete remission [6]. Therefore, it is necessary to explore new diagnostic methods for the detection and early intervention of thyroid cancer.

Autophagy is a reaction of cells to changes in internal and external environmental pressure [7–9]. It is widely present in eukaryotic cells and is a mechanism for organisms to purify their excess or damaged organelles during their development and aging. Autophagy can be divided into three types according to the different ways of transporting substrates into the lysosomal cavity: macroautophagy, microautophagy and molecular chaperone-mediated autophagy. Generally, autophagy refers to macroautophagy [10]. Autophagy is a method of self-renewal of cells, but excessive autophagy can lead to cell death, namely, autophagic cell death, also known as type II programmed cell death [11, 12]. When apoptosis is inhibited, autophagy plays a role in promoting cell death [13]. Autophagy plays a dual role in promoting and inhibiting the occurrence and development of tumors. However, the specific mechanisms are not completely clear. In the early stage of many cancers, autophagy can inhibit the transformation and growth of cancer cells. Additionally, it can meet the metabolic needs of rapid growth by degrading and restoring the components of damaged or aging organelles, which ultimately induces the exaggerated proliferation of cancer cells [14–17]. Studies have shown that autophagy is closely related to the growth and development of thyroid cancer. For example, Beclin l, which is considered to be a homologous gene of AT96 and a tumor suppressor gene in mammals, is a very important autophagy regulatory gene. The incidence of tumors in mice with Beclin 1 deletion was increased [18]. However, studies in 2013 showed that Beclin 1 expression was significantly higher in papillary thyroid carcinoma and metastatic lymph nodes than in normal tissues, suggesting that the expression of Beclin-1 in papillary thyroid carcinoma is related to tumor occurrence and lymph node metastasis [19]. In 2014, Zhang et al. found that in undifferentiated thyroid cancer, miR-30d can combine with the 3' UTR region of Beclin 1 to inhibit its expression, thus reducing the level of Beclin 1-mediated autophagy, ultimately leading to undifferentiated thyroid cancer being more sensitive to cisplatin [20]. In other words, Beclin1 can initiate autophagy to help undifferentiated thyroid cancer cells escape the apoptosis caused by cisplatin, thereby promoting the development of thyroid cancer.

In view of these conflicting results, we attempted to explore the potential value of autophagy in THCA by integrating the full set of autophagy-related genes (ARGs) and corresponding gene expression with clinical data obtained from The Cancer Genome Atlas (TCGA) database. We first identified the differentially expressed autophagy-related genes (DEARGs) in THCA and constructed a risk prediction model. Then, Lasso regression and Cox regression analysis were used to optimize the model, and the DEARGs related to overall survival (OS) were selected. We used these DEARGs to establish a Cox regression model and evaluated the specificity and sensitivity of the model by receiver operating characteristic (ROC) curve analysis. Our data show that the model can accurately predict the prognosis of patients. These findings also provide an effective multidimensional strategy based on biomarkers for the prognosis prediction of patients with THCA.

## RESULTS

### Flow chart of this study

**Figure 1:**
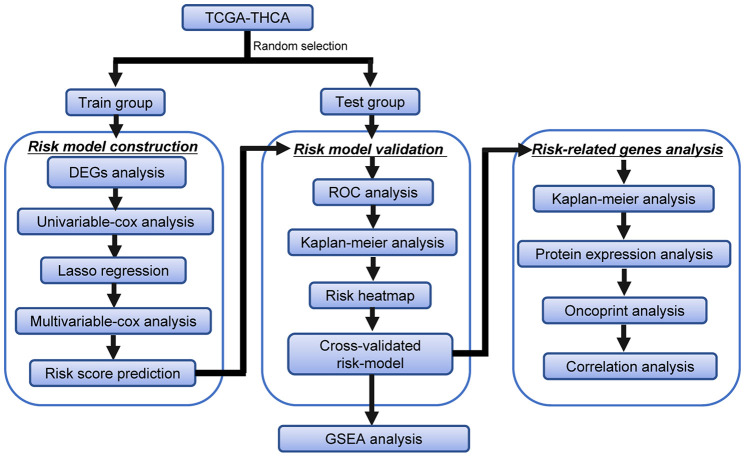
**Flowchart of the identification of the survival-related autophagy gene signature in THCA.**

The detailed workflow of the risk model construction and downstream analysis is shown in Figure 1. We first found differentially expressed ARGs in THCA. Then, these DEARGs were identified in the training data set to build a specific risk model. The risk model was further verified and optimized in the testing data set. Time-dependent ROC analysis was used to test the predictive ability of the risk model. Finally, Kaplan-Meier analysis, protein expression analysis, OncoPrint analysis and correlation analysis were performed on the genes in the risk model.

### Differential expression and functional annotation of ARGs in THCA

**Figure 2:**
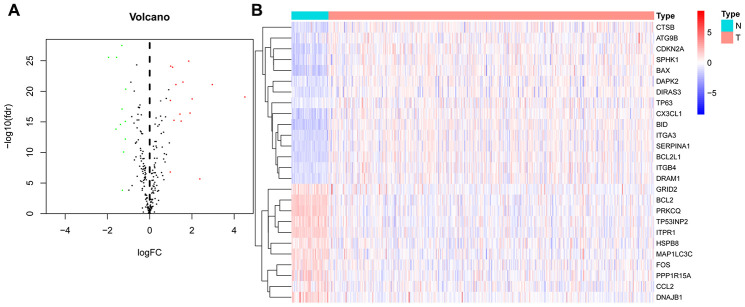
**Differentially expressed autophagy-related genes (DEARGs) in thyroid carcinoma (THCA) and nontumor samples.** (**A**) The volcano map of 232 ARGs. The red dots indicate genes with high expression, and the green dots represent genes with low expression. (**B**) Clustered heatmap of DEARGs in THCA and normal thyroid tissues.

**Figure 3:**
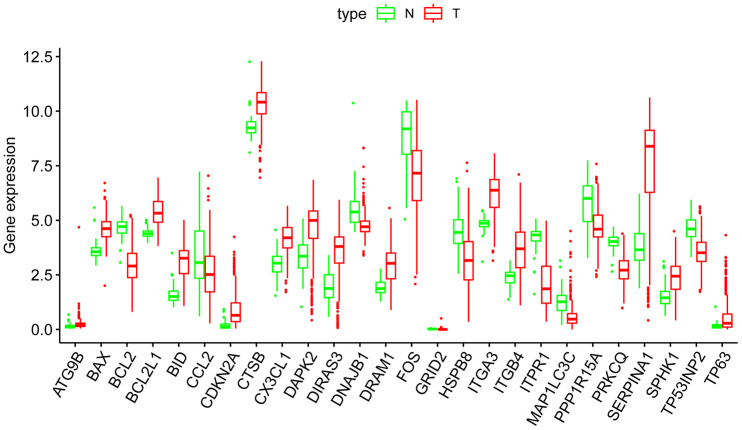
**Boxplots of the expression levels of 26 autophagy-related genes (ARGs) in THCA and normal thyroid tissues.** The red box plots above the corresponding gene name represent the expression in THCA, whereas the green box plots represent the expression in normal thyroid tissues.

**Figure 4:**
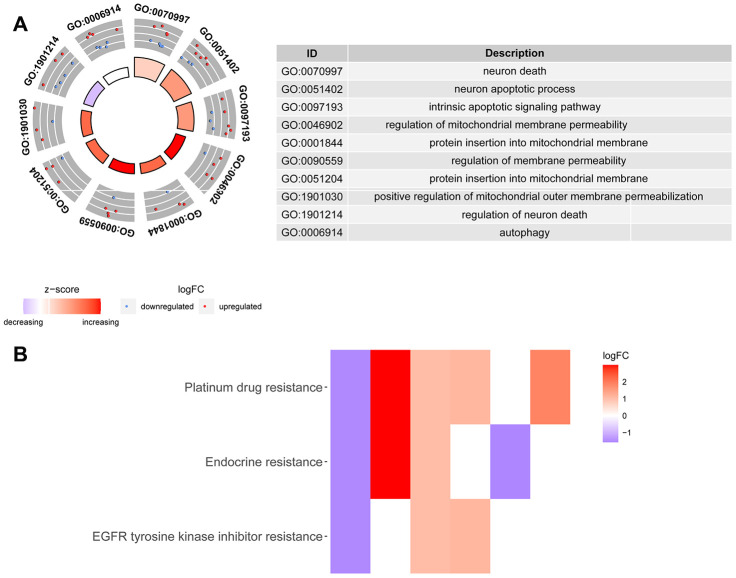
**Gene Ontology and KEGG enrichment analysis of DEARGs.** (**A**) Results of Gene Ontology (GO) functional annotation analysis. The outer circle shows a scatter plot for each term of the assigned genes based on the logFC. Red circles display upregulated pathways, and blue circles show downregulated pathways. (**B**) Results of Kyoto Encyclopedia of Genes and Genomes (KEGG) pathway enrichment analyses of the autophagy-related genes. Heatmap of the relationship between the ARGs and pathways. The color of each block depends on the logFC values.

We downloaded the mRNA expression data and corresponding clinical information of 510 THCA tissue samples and 58 nontumor samples from the TCGA database (Table 1, Supplementary Table 1). After extracting the expression values of 232 ARGs, we obtained differentially expressed ARGs, and the expression patterns of the DEARGs in THCA and nontumor tissues were depicted in volcano and heat maps (Figure 2). In THCA, 26 differentially expressed genes were found, of which 11 genes were downregulated in tumor tissues and 15 genes were upregulated (Figure 3). Then, we performed functional enrichment analysis of the DEARGs, which is helpful for understanding the biological processes of these genes. Figure 4 and Supplementary Table 2 summarize the GO term and KEGG pathway enrichment of these genes. In THCA, we found that the most abundant GO items in the biological process category were neuron death, neuron apoptotic process and intrinsic apoptotic signaling pathway. The most enriched cellular components were the mitochondrial outer membrane, autophagosome and organelle outer membrane. In terms of molecular functions, the genes were mostly enriched in BH domain binding, death domain binding and protease binding (Figure 4A). In addition, in the KEGG pathway enrichment analysis of the differentially expressed ARGS, we found that these genes were significantly correlated with platinum drug resistance, endocrine resistance and EGFR tyrosine kinase inhibitor resistance (Figure 4B).

**Table 1:** Clinicopathological parameters of THCA patients in the TCGA database.

**Clinical parameters**	**Variable**	**Total(507)**	**Percentages(%)**
**Age**	<=65	436	86.0%
	>65	71	14.0%
**Pathological stage**	Stage I	285	56.2%
	Stage II	52	10.3%
	Stage III	113	22.3%
	Stage IV	55	10.8%
	Unknow	2	0.4%
**T**	T1	144	28.4%
	T2	167	32.9%
	T3	171	33.7%
	T4	23	4.5%
	TX	2	0.4%
**M**	M0	283	55.8%
	M1	9	1.8%
	MX	214	42.2%
	Unknow	1	0.2%
**N**	N0	231	45.6%
	N1	226	44.6%
	NX	50	9.9%
**Survival status**	Dead	16	3.2%
	Alive	491	96.8%

Abbreviations: T, Tumor; M, Metastasis; N, Node (regional lymph node).

**Table 2:** The five selected autophagy-related genes.

**Id**	**Coef**	**HR**	**HR.95L**	**HR.95H**	**P value**
CX3CL1	-1.0853	0.3378	0.1132	1.0081	0.0517
CDKN2A	1.2299	3.4208	1.4762	7.9268	0.0041
ATG9B	-0.7449	0.4748	0.2112	1.0674	0.0715
ITPR1	0.8326	2.2993	1.1854	4.4599	0.0138
DNAJB1	0.8631	2.3705	1.2793	4.3923	0.0061

Abbreviations: HR, hazard ratio; HR.95 L/H, 95 % confidence interval of the hazard ratio.

### Construction and verification of the THCA risk model

**Figure 5:**
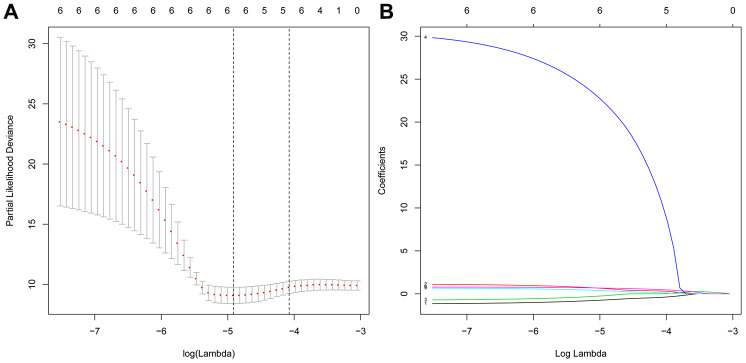
**Screening of the optimal ARGs with prognostic potential by Lasso regression.** (**A**) Screening of the optimal parameter (lambda) at which the vertical lines were drawn. (**B**) Lasso coefficient profiles of the six ARGs with nonzero coefficients determined by the optimal lambda.

**Figure 6:**
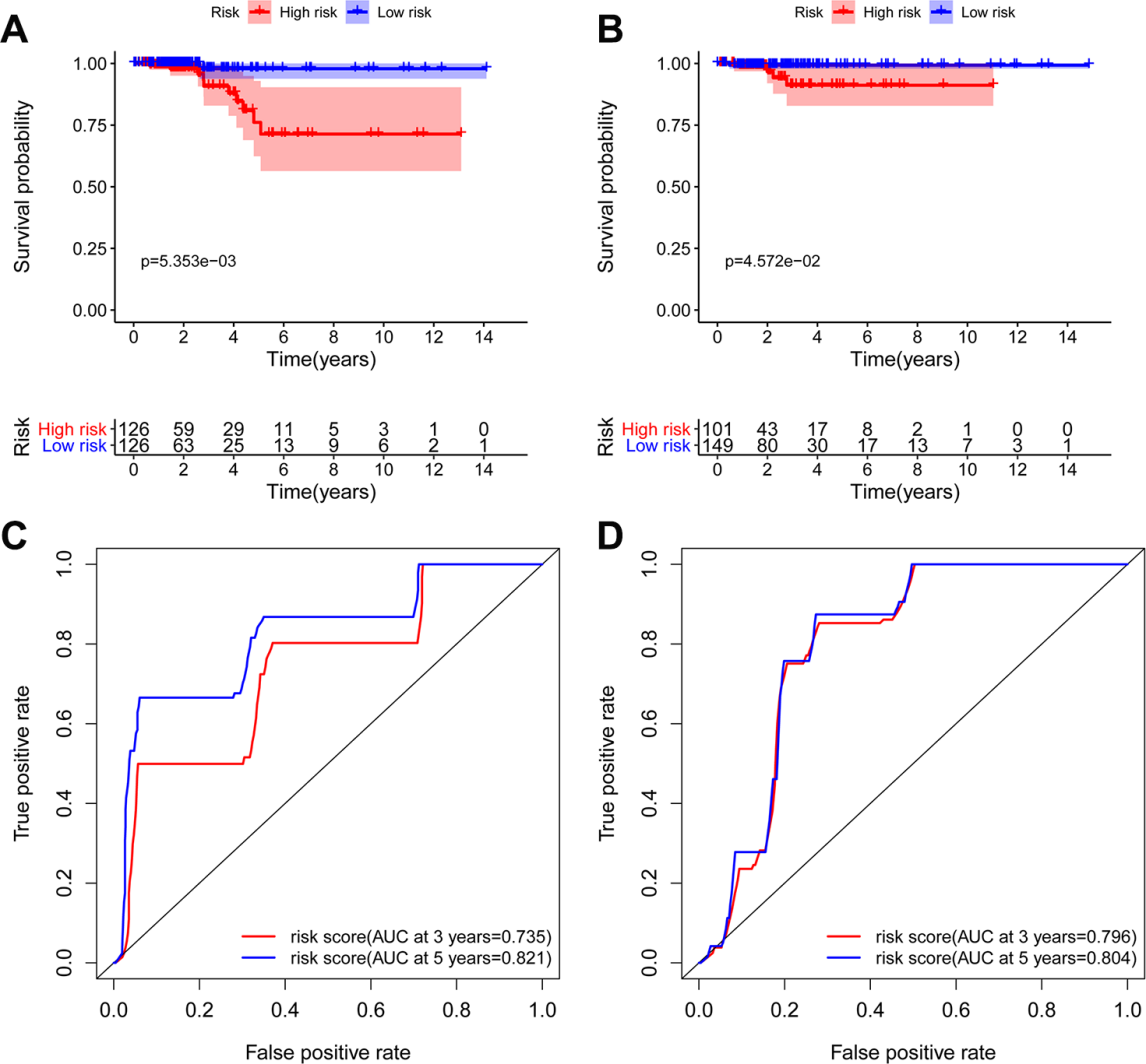
**Kaplan-Meier and ROC analyses in the training and testing groups.** Kaplan-Meier plot of the high-risk (red) and low-risk (blue) THCA patients in the training group (**A**) and testing group (**B**). The 3-year (red) and 5-year (blue) ROC curves of THCA patients in the training group (**C**) and testing group (**D**).

**Figure 7:**
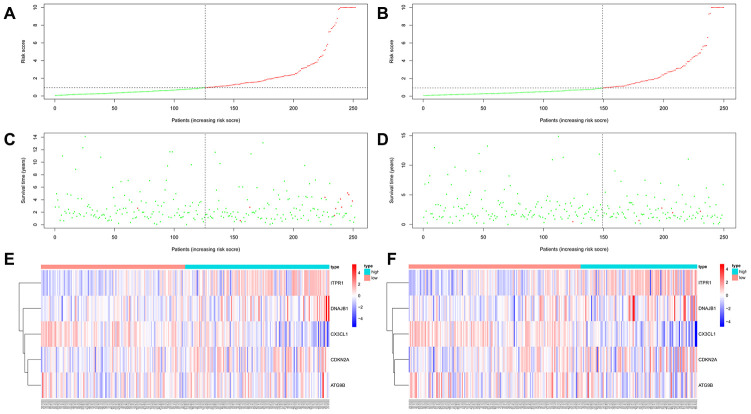
**Autophagy-related prognostic characteristics in patients with thyroid carcinoma.** Risk score distribution of THCA patients with different risks (low, green; high, red) in the training group (**A**) and testing group (**B**). Dot plots showing the survival time and risk score in the training group (**C**) and testing group (**D**). Heatmap of the expression profiles of the 5 key genes in the training group (**E**) and testing group (**F**).

To explore the relationship between ARGs and prognosis, we established a risk model in patients with thyroid cancer. Initially, univariate Cox regression analysis was performed to obtain genes significantly related to prognosis, and then lasso regression and multivariate Cox regression were used to generate the final prognostic model (Figure 5, Table 2). After establishing the risk model, the patients were divided into a high-risk group and a low-risk group, and then Kaplan-Meier survival analysis was conducted on the training set and testing set. The results showed that patients with high risk scores had significantly worse overall survival times than those with low risk scores in the THCA dataset (Figure 6A and 6B). Among THCA patients, we successfully obtained a 5-gene model (*CX3CL1, CDKN2A, ATG9B, ITPR1 and DNAJB1*). Using this model to predict the risk score of each patient, we determined that *CDKN2A, ITPR1 and DNAJB1* were positive risk-related genes, while *CX3CL1* and *ATG9B* were negative risk-related genes. The area under the ROC curve (AUC) in the training and testing sets was significant (3-year AUC, 0.735 vs 0.796; 5-year AUC, 0.821 vs 0.804). This model can accurately predict the OS of THCA patients (Figure 6C and 6D). In addition, we ranked all THCA patients according to their risk scores to analyze the survival rate distribution. The scatterplot shows the survival status of patients with different risk scores, and the mortality rate of patients increases with increasing risk scores. The heat map showed that the expression of ARGs was related to an increase in the risk score of patients (Figure 7A–7F).

### The prognostic risk model of THCA patients was independently related to OS

**Figure 8:**
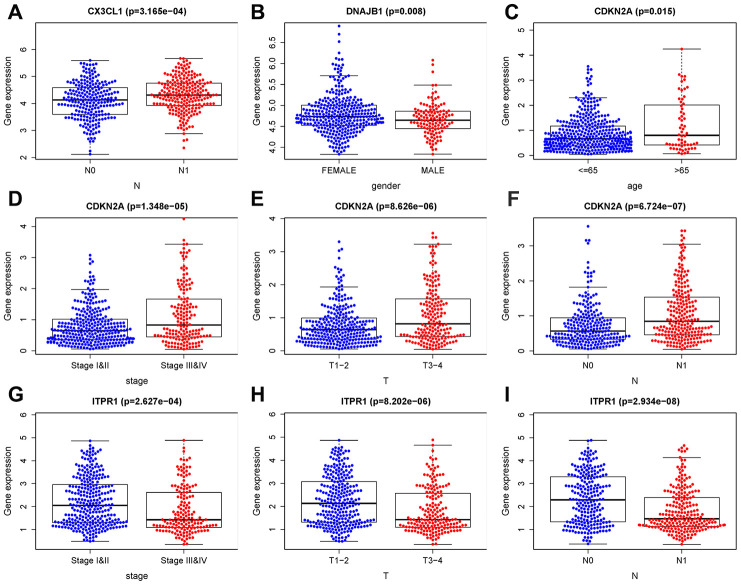
**Clinical correlation analysis of ARGs.** (**A**) *CX3CL1* expression was negatively correlated with pathological N stage. (**B**) The expression of *DNAJB1* was positively correlated with sex. (**C–F**) *CDKN2A* expression was negatively correlated with age, histological grade, and pathological T and N stages. (**G–I**) *ITPR1* expression was positively correlated with histological grade and pathological T and N stages. T: tumor, N: lymph node, M: metastasis.

Univariate Cox regression and multivariate Cox regression were used to analyze the correlations of OS with factors such as age, histological grade, pathological stage, and risk score of patients with THCA. Univariate Cox regression analysis showed that age, histological grade, pathological T stage, and risk score of patients with THCA were related to OS (P <0.05), while multivariate Cox analysis showed that age and risk score were related to OS in THCA patients (P <0.05) (Table 3). At the same time, we also analyzed the correlation between gene expression and clinical characteristics, as shown in Figure 8. CX3CL1 expression was negatively correlated with pathological N stage. The expression of DNAJB1 was positively correlated with sex. CDKN2A expression was negatively correlated with age, histological grade, and pathological T and N stages. ITPR1 expression was positively correlated with histological grade and pathological T and N stages. These results indicate that the established prognostic model and genes can be used to predict the OS of THCA patients.

**Table 3:** Univariate and multivariate Cox regression analyses of OS in THCA.

**Id**	**uniForest**		**multiForest**	
	**HR (95% CI)**	**P**	**HR(95% CI)**	**P**
Age	1.1487 (1.0755-1.2268)	0.0000	1.1792 (1.0740-1.2948)	0.0005
Gender	0.8983 (0.1855-4.3496)	0.8939	1.0901 (0.1747-6.8034)	0.9264
Stage	2.7939 (1.4286-5.4639)	0.0027	1.0130 (0.1371- 7.4863)	0.9899
T	2.3948 (1.0828-5.2963)	0.0311	2.0166 (0.3740-10.8724)	0.4145
M	2.3350 (0.2853-19.1078)	0.4291	2.2176 (0.1806-27.2242)	0.5336
N	2.1207 (0.5295-8.4932)	0.2883	0.4617 (0.0579-3.6835)	0.4658
riskScore	1.0201 (1.0006-1.0400)	0.0435	1.0322 (1.0013-1.0640)	0.0409

Abbreviations: OS, Overall survival; HR, hazard ratio; T, Tumor; M, Metastasis; N, Node(regional lymph node).

### Comprehensive analysis of genes in the autophagy prognostic model

**Figure 9:**
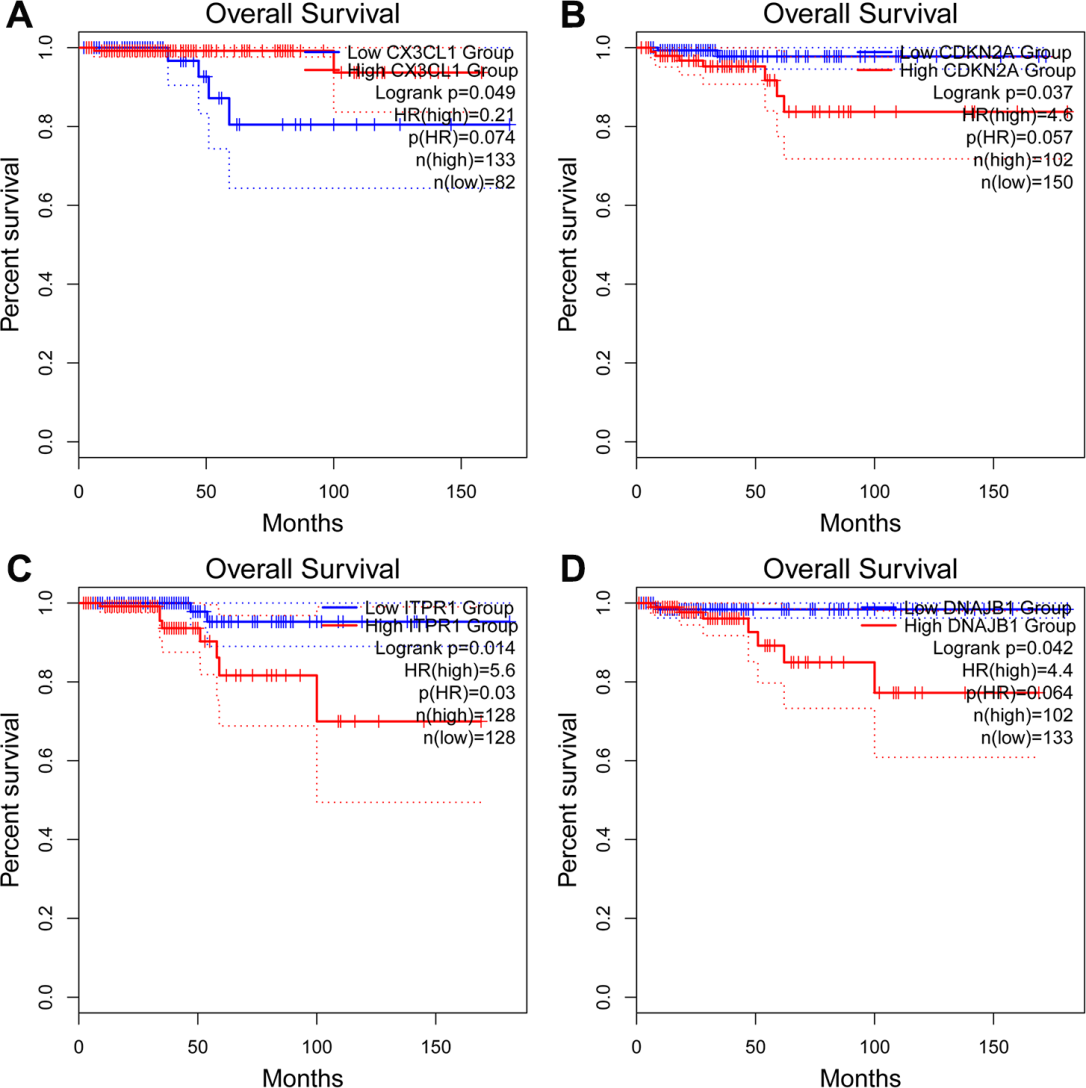
**Kaplan-Meier analyses of ARGs in the risk model.** Kaplan-Meier analyses of (**A**) *CX3CL1*, (**B**) *CDKN2A*, (**C**) *ITPR1*, and (**D**) *DNAJB1*. The statistical significance was determined by *the log-rank* test.

**Figure 10:**
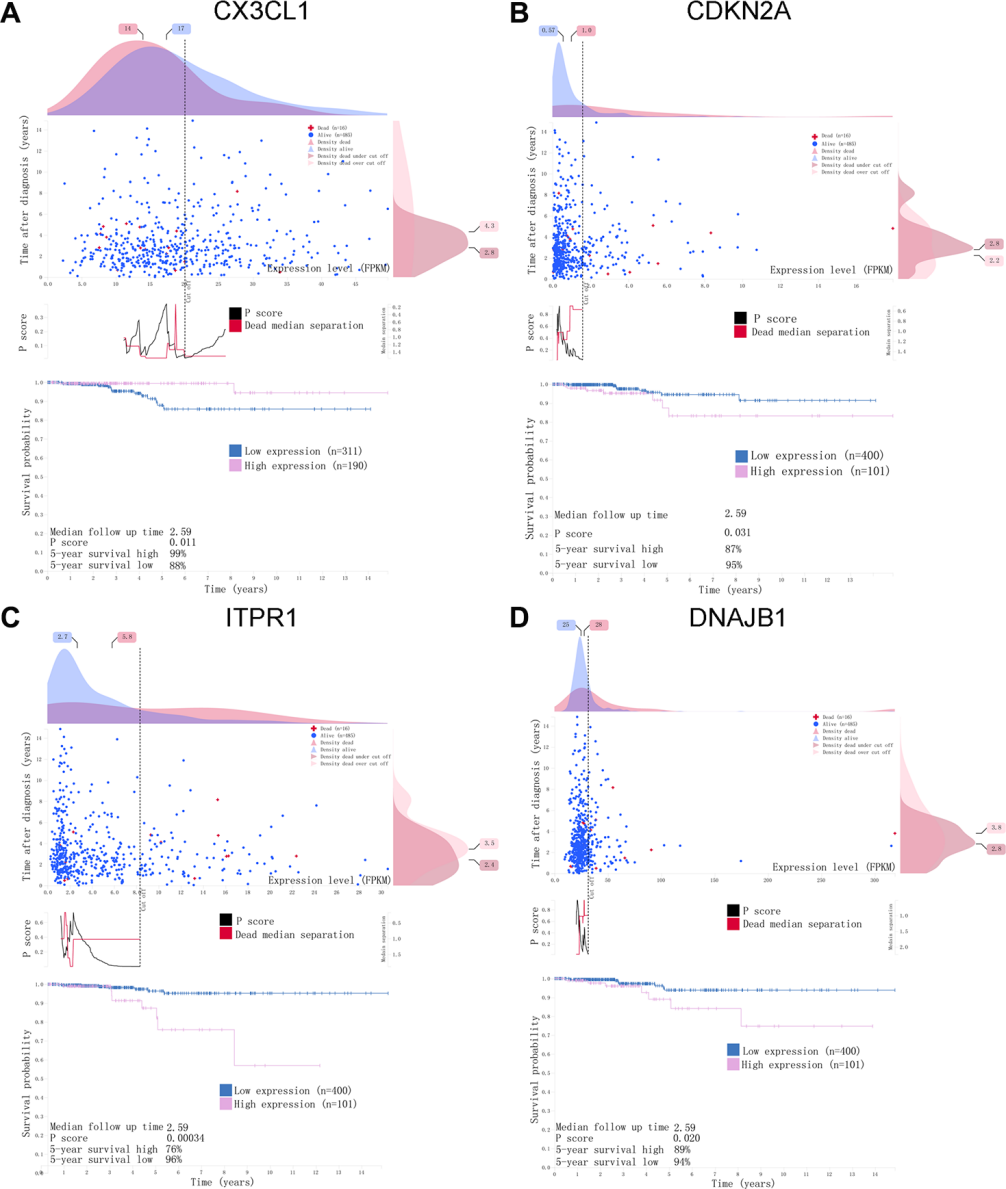
**A horizontal distribution of events and gene expression in the Human Protein Atlas (HPA) database.** Kaplan-Meier survival curves of (**A**) *CX3CL1*, (**B**) *CDKN2A*, (**C**) *ITPR1*, (**D**) *DNAJB1* in THCA. The pink line indicates the high expression group, while the blue line indicates the low expression group. P <0.05 was set as the cutoff criterion.

**Figure 11:**
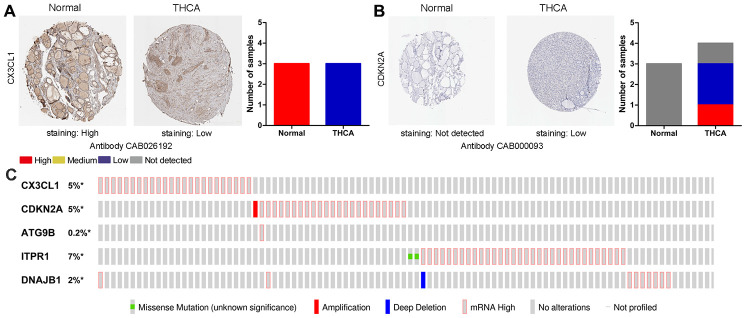
**Immunohistochemistry (IHC) results and mutations in prognosis-related ARGs.** (**A**, **B**) The protein expression of *CX3CL1* and *CDKN2A* was determined by immunohistochemistry using the indicated antibodies in the HPA database, and the staining strengths were annotated as Not detected, Low, Medium and High. The bar plots indicate the number of samples with different staining strengths in the HPA database. (**C**) OncoPrint showing the copy number alterations and mRNA expression alterations of the 5 ARGs in the autophagy prognostic model.

**Figure 12:**
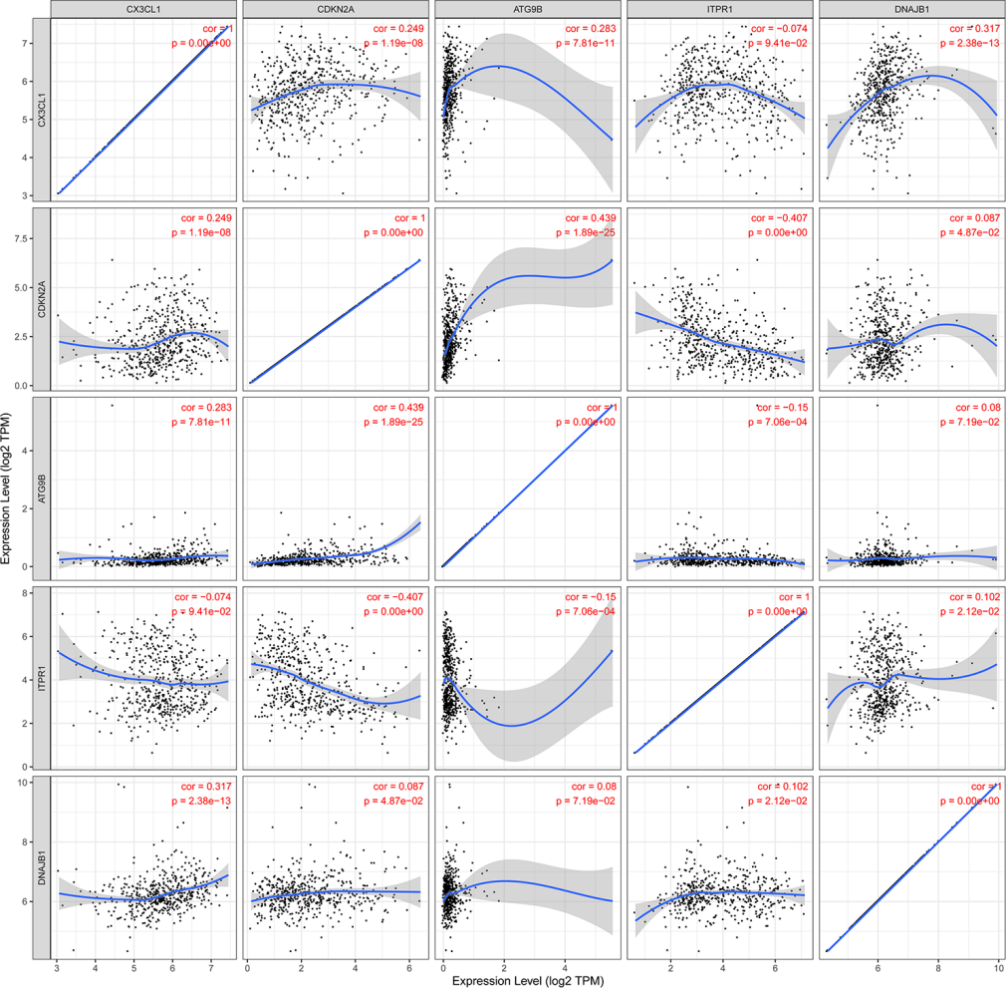
**The relationships between the expression levels of the 5 ARGs in the autophagy prognostic model.** Cor: Correlation coefficient. The value range of the correlation coefficient is (-1, 0) or (0, 1). When the value range is (-1, 0), it indicates a negative correlation; when the value range is (0, 1), it indicates a positive correlation.

**Figure 13:**
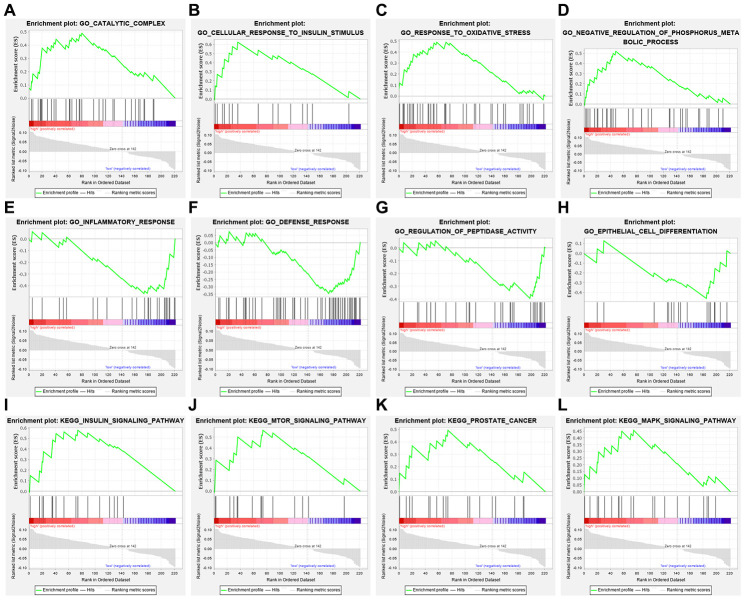
**Gene set enrichment analysis of genes in high-risk and low-risk patients with THCA.** (**A**–**D**) GO enrichment analysis results in the high-risk group. (**E**–**H**) GO enrichment analysis results in the low-risk group. (**I**–**L**) KEGG enrichment analysis results in the high-risk group.

For the risk model, we identified a total of 5 genes and then further evaluated the prognostic value of the selected genes in other databases. The genes were analyzed by Kaplan-Meier in the GEPIA database. The results showed that *CDKN2A, ITPR1* and *DNAJB1* were negatively correlated with OS in THCA, while high expression of *CX3CL1* indicated a good prognosis (Figure 9). In general, the results of the Kaplan-Meier analysis were consistent with the results of the univariate Cox analysis, which indicates that most of the genes are significant in the risk model and have strong predictive power. Next, we analyzed the protein expression pattern of the genes in the risk model through the HPA database (Figures 10 and 11A, 11B). The results showed that *CX3CL1* protein was less expressed in normal thyroid tissue but moderately expressed in thyroid cancer tissue (Figure 11A). *CDKN2A* was not detected in normal tissues but was expressed at low levels in tumor tissues (Figure 11B). These results were consistent with most mRNA levels we observed previously. There were no data on *ATG9B* in the Human Protein Atlas database. Then, we used the cBioPortal database to examine the CNV and mRNA expression changes of these genes (Figure 11C). The results showed that CNVs were involved in the mRNA expression changes of these genes. It is worth noting that *CDKN2A*, *ITPR1* and *CX3CL1* showed the highest CNVs and mRNA expression changes in the entire analysis sample, which may indicate that CNVs are the main driving forces for the mRNA expression changes of these genes. The correlation analysis of the five selected genes in the TIMER database shows that most genes were closely related in mRNA expression (Figure 12). Because we found that high-risk and low- risk patients have significant prognostic differences in OS, we next used the GSEA method to explain the enriched features and pathways between high-risk and low-risk patients. In the GSEA enrichment results, we observed that signals such as “*RESPONSE_TO_OXIDATIVE_STRESS*”, “*MTOR_SIGNALING_PATHWAY*”, and “*MAPK_SIGNALING_PATHWAY*” were enriched in the high-risk group, while the low-risk group showed enrichment in “*INFLAMMATORY_RESPONSE*”, “*DEFENSE_RESPONSE*”, “*REGULATION_OF_PEPTIDASE_ACTIVITY*”, and “*EPITHELIAL_CELL_DIFFERENTIATION*” (Figure 13). Some studies have shown that these signals are related to the occurrence and development of thyroid cancer. In summary, the GSEA results suggest that autophagy-related signals are related to the development and progression of THCA.

## DISCUSSION

Thyroid cancer is the most common malignant tumor of the endocrine system, with a good prognosis in general. However, for papillary thyroid cancer, undifferentiated thyroid cancer and medullary thyroid cancer with radiation resistance, traditional antitumor treatment cannot achieve satisfactory results [21, 22]. In recent years, with increasing research on autophagy in the occurrence and development of thyroid cancer, researchers have gradually focused on autophagy as a target for the treatment of thyroid cancer that is not sensitive to conventional therapies. Although there is an increasing number of studies on the relationship between autophagy and thyroid cancer, how to apply autophagy to the clinical treatment of thyroid cancer still needs deeper basic and clinical research to clarify the internal mechanisms of autophagy and the occurrence and development of thyroid cancer in order to provide a sufficient theoretical basis for the further use of autophagy as a new target for the treatment of thyroid cancer. High-throughput sequencing technology can recognize various biomarkers at the gene level that are closely related to the survival of patients. Here, we used TCGA-THCA data to determine autophagy genes related to prognosis by bioinformatics analysis and developed a model for the prognosis of THCA.

To the best of our knowledge, this is the first study to combine the whole set of ARGs with THCA data to explore and verify the potential value of ARGs in THCA. In this study, we explored the expression of ARGs in the TCGA database to find molecular biomarkers related to the diagnosis, treatment, and prognosis of THCA patients. We first screened the DEARGs between THCA and nontumor tissues. Considering that these genes may be closely related to the occurrence of THCA, we conducted GO and KEGG analyses of these genes. In fact, most enrichment pathways were autophagy-related pathways. Interestingly, other pathways were also found to be enriched, including neuron apoptotic process, intrinsic apoptotic signaling pathway, etc. Regarding KEGG pathways, we found that the genes were mainly enriched in signaling pathways related to drug resistance, such as platinum drug resistance, endocrine resistance and EGFR tyrosine kinase inhibitor resistance. The role of autophagy in tumor drug resistance has also become the focus of recent research. Many previous reports have shown that autophagy is the main mechanism for removing mitochondria and dysfunctional proteins damaged by ROS [23–25]. Therefore, the excessive occurrence of autophagy is an important factor for the resistance of tumor cells to cytotoxic drugs. In a variety of tumor models, autophagy can remove abnormal proteins, organelles and ROS to play a role in drug resistance [26–28]. A study found that high migration rate family protein B1 (HMGB1) and other prototype cell damage-related molecular patterns (DAMPs) can increase the clearance rate of organelles in tumor cells that are damaged by antitumor drugs by upregulating autophagy, thereby enhancing tumor drug resistance [29]. Kong et al. found that in both wild-type and mutant p53 ovarian cancer cells, tumor MDR with autophagy as the self-protection mechanism can be reversed by the inhibition of autophagy. In addition, MDR mutant ovarian cancer cells can be killed by activating the autophagic cell death process, and the inhibition of autophagy can also reverse MDR in the p53 wild-type ovarian cancer cell line [30]. EGFR is a transmembrane glycoprotein with tyrosine kinase activity that is widely expressed in human epithelial cells. Current research shows that EGFR is highly expressed in a variety of epithelial tumors, especially in liver cancer, colorectal cancer, breast cancer and so on, and its expression level is closely related to tumor metastasis and prognosis [31, 32]. In thyroid cancer tissues, EGFR is highly expressed and related to lymph node metastasis, tumor histology, and clinical stage, which may indicate the aggressiveness of tumor, and its expression level is one of the important markers in clinical surgery and prognosis [33]. EGFR participates in mitochondrial biosynthesis, maintains mitochondrial stability, and prevents apoptosis. Autophagy can provide an alternative energy source when cells are damaged by chemotherapy and radiotherapy. The inhibition of autophagy in EGFR-overexpressing cells is expected to be a new treatment. The study found that cells with a high expression of EGFR have reduced autophagy flow, and these cells normally depend on autophagy for proliferation and survival [34]. After EGFR siRNA transfection, prostate cancer cell autophagy activity increased [35]. EGFR inhibitors have also been found to induce autophagy in non-small cell lung cancer (NSCLC) and many other tumor cells [36]. In the transplanted tumor model of head and neck squamous cell carcinoma (HNSCC), EGFR was negatively correlated with the expression of the autophagy marker LC3B, indicating that EGFR signaling is involved in the regulation of autophagy function [37]. In addition, EGFR also inhibits autophagy by maintaining a highly activated PI3K-AKT-mTOR signaling pathway [38]. All the above studies showed that the expression of EGFR was closely related to the expression of autophagic markers and the autophagic activity of cells. Then, we established the model by univariate Cox regression and lasso regression and finally identified five OS-related risk genes (CX3CL1, CDKN2A, ATG9B, ITPR1 and DNAJB1). We further constructed a risk model and proved that this model can provide accurate predictions of the prognosis of THCA patients in the validation group and training group. In addition, multivariate Cox regression analysis of the prognostic model and other clinical parameters showed that the model could independently predict the prognosis of THCA patients. Moreover, GSEA showed that the mTOR and MAPK signaling pathways were overactivated in high-risk patients, suggesting that tumors in the high-risk group may have a higher potential for proliferation, migration and invasion [39]. The enrichment analysis of the low-risk group indicated that the increase in peptidase activity, inflammatory response, defense response and epithelial differentiation ability all inhibited the progression of tumors.

There are still some limitations in this study. The main limitation is that the data used in our study came from multiple public databases. In addition, the clinical significance of these findings is challenging and unclear at present, and the mechanisms of ARGs regulating the occurrence and development of THCA need further study. In conclusion, based on the comprehensive analysis of ARG expression profiles and related clinical features, this study established a prognostic ARG-based risk model. The autophagy genes in this model provide new targets for the treatment and intervention of thyroid cancer. However, local clinical trials are needed to further validate the findings of this study to help personalize clinical treatment.

## MATERIALS AND METHODS

### Acquisition of datasets

In this study, 232 autophagy-related genes were downloaded from human autophagy database (HADB, http://www.autophagy.lu/index.html), and clinicopathological parameters and RNA sequencing results (FPKM) of THCA were obtained from TCGA data portal (https://portal.gdc.cancer.gov/). The expression data were Convert to TPM and perform downstream analysis.

### Analysis and screening of differential expression of args

The Wilcox test in R (version 3.5.3, https://www.R-project.org/) was used to estimate the differential expression of ARG between THCA and non-tumor specimens. The genes with at least 2-fold variation and P value less than 0.05 were selected as ARG with significant differential expression (DEARG). Then a series of gene function enrichment analysis was carried out to discover the main biological characteristics of these genes. The clusterProfiler package in R is used to identify enriched GO and KEGG. Next, we integrate the expression data of DEARGs with corresponding clinical information. Finally, the data is randomly divided into training group and testing group for subsequent verification. The expression data of 26 ARGs in the training group were analyzed by univariate Cox regression, and DEARGs significantly correlated with survival rate were obtained (P<0.05). Then lasso analysis was used to eliminate the false positive parameters caused by over fitting. Finally, Cox proportional risk regression was used to establish OS prognostic risk model.

### Construction and verification of risk prediction model based on DEARGs

We conducted multivariate Cox regression analysis to determine five prognoses args and their coefficients, and then constructed a prognosis model. In the training group and the testing group, the risk score of each patient was calculated according to the regression coefficient of single gene and the expression value of each gene. The calculation formula is as follows. The calculation of the risk score based on the ARGs model was conducted as follows: $\text{Riskscore}=\sum_{\text{i}=1}^{\text{n}}\text{vi}\times \text{ci}$ (the vi is the expression value of gene i, ci represents the regression coefficient of gene i in the multivariate Cox regression analysis, and n represents the number of independent indicators). Risk score is a measure of prognosis risk of each THCA patient. We used the median risk score of the training group as the boundary to divide THCA patients into a high-risk group and a low-risk group, draw a Kaplan-Meier (KM) survival curve, and use a log-rank test to assess the difference in overall survival rates between the two groups to determine Statistical significance. In addition, we also generate receiver operating characteristic (ROC) curves to determine the accuracy of the prediction model.

### Comprehensive analysis of ARGs in risk model

Using the GEPIA database (http://GEPIA.cancer-pku.cn/), Kaplen-Meier analysis of ARGs in risk models was performed. By comparing the immunohistochemical staging images in the human protein atlas database (http://www.proteinAtlas.org/), the protein expression of the selected ARGs was analyzed. On the basis of the staining intensity, it was labeled as undetected, low, medium and high. The cbioProtal database (http://www.cbioportal.org/) was used to further analyze the five ARGs in the risk model to assess changes in copy number and mRNA expression. Perform correlation analysis on the screened ARGs in TIMER database, and calculate the Pearson correlation coefficient between each gene pair. Finally, in order to explore the characteristics and ways of enrichment in the predicted high- and low-risk populations, gene set enrichment analysis (GSEA) was carried out. Using GSEA, this study examined whether the characteristics of activation/inhibition genes were abundant in high-risk and low-risk patients. The enrichment degree of the predefined signature and KEGG path is calculated using standardized enrichment score (NES) and standardized P value. Terms with | NE | > 1 and P < 0.05 are considered to be significantly.

### Statistical analysis

R software (version = 3.5.3) was used for all statistical analysis. Cox regression analysis was used to screen survival-related ARGs. Lasso regression analysis was used to eliminate the highly relevant ARGs and prevent overfitting of the model. Kaplan Meier curve was drawn to show the difference of overall survival rate between the two groups, and the log rank test was carried out to determine the significance of the difference. The ROC curve and the area under the curve (AUC) are used to evaluate the performance of the model. ROC curve was drawn by using the survival ROC package in the R software. The AUC value greater than or equal to 0.70 is the effective prediction value, and the AUC value greater than or equal to 0.6 is the acceptable prediction value. Statistical significance was defined as P < 0.05.

## Supplementary Material

Supplementary Table 1

Supplementary Table 2
